# Eating garlic and onion: a matter of life or death

**DOI:** 10.1038/sj.bjc.6601918

**Published:** 2004-06-15

**Authors:** A A Izzo, R Capasso, F Capasso

**Affiliations:** 1 Department of Experimental Pharmacology, University of Naples Federico II, via D. Montesano 49, Naples 80131, Italy

**Sir,**

Diet is thought to be one of the most important contributing factors to cancer risk (Bingham and Riboli, 2004). Among the dietary improvements that can reduce the risk of cancer, Stein and Colditz (2004) highlighted the promising relationship between prostate cancer and tomatoes intake. Here, we wish to draw attention to *Allium* vegetables (i.e. garlic and onion), which, on the basis of epidemiological studies, have been showing promising signs of possessing chemopreventive activity in patients with prostate cancer.

Garlic (*Allium sativum*) and onion (*Allium cepa*) are among the oldest of all cultivated plants and now used as a food and for medical purpose (e.g. garlic for hypercholesterolaemia and hypertension) (Capasso *et al*, 2003). Site-specific, case–control studies and cohort studies suggest a preventive effect of *Allium* vegetables consumption against stomach, colorectal and prostate cancer, although evidence for a protective effect against cancer at other sites, including the breast, is still lacking (Ernst, 2000). Most notably, a population-based, case–control study, performed on 238 patients with prostate cancer and 471 male control subjects, investigated the association between intake of *Allium* vegetables and the risk of prostate cancer (Hsing *et al*, 2002). It was found that men in the category of highest intake of *Allium* vegetables (>10 g day^−1^) had a statistically significant lower risk of contracting prostate cancer than did those in the category of lowest intake (2.2 g day^−1^). The reduced risk of prostate cancer associated with *Allium* vegetables was independent of body size, intake of other foods and total calorie intake, and was more pronounced for men with localized than with advanced prostate cancer (Hsing *et al*, 2002).

Experimental studies have shown that the chemopreventive activity of *Allium vegetables* is related to their content of organosulphur compounds (OSCs). Although how these compounds achieve chemoprevention is not fully understood, several modes of action have been proposed (Knowles and Milner, 2001; Griffiths *et al*, 2002; Rahman, 2003; Thomson and Ali, 2003). These include: (i) effect on drug metabolising enzymes (i.e. induction of phase II detoxification enzymes, including glutathione transferases, quinine reductase, epoxide hydrolase and glucuronosyl transferase, that inactivate toxic substances and facilitate their excretion); (ii) antioxidant activity (garlic preparations exhibit radical scavenging activity and decrease lipid peroxidation, which is relevant in the light of the observation that tumour promotion may involve oxygen radicals); (iii) tumour growth inhibition that has been documented in several carcinoma cell lines, including prostate carcinoma cells; (iv) induction of apoptosis, which coincides with an increase in the percentage of cells blocked in the G_2_/M phase of the cell cycle (possibly through a depression in p34^cdc2^ kinase); and (v) effective stimulation of the immune response (OSCs stimulates proliferation of lymphocytes and macrophage phagocytosis, induce the infiltration of macrophages and lymphocytes in transplanted tumours, induce splenic hypertrophy, stimulate release of interleukin-2, tumour necrosis factor-*α* and interferon-*γ*, enhance natural killer cell, killer cell and lymphokine-activated killer cell activity).

In conclusion, promising data from clinical studies suggest that the regular consumption of garlic and onion may prevent prostate cancer. *Allium*-derived organosulphoric compounds may be responsible, at least in part, for the anticarcinogenic activity. The unique property of garlic and onion may be clinically important, because their daily intake for a prolonged period is expected to be free of risk.
